# The Swedish study of Irrigation-fluid temperature in the evacuation of Chronic subdural hematoma (SIC!): study protocol for a multicenter randomized controlled trial

**DOI:** 10.1186/s13063-017-2194-y

**Published:** 2017-10-11

**Authors:** Andreas Bartley, Asgeir S. Jakola, Jiri Bartek, Jimmy Sundblom, Petter Förander, Niklas Marklund, Magnus Tisell

**Affiliations:** 1 Department of Neurosurgery, Sahlgrenska University Hospital, Blå stråket 5, 41345 Gothenburg, Sweden; 2 Institute of Neuroscience and Physiology, Department of Clinical Neuroscience, University of Gothenburg, Sahlgrenska Academy, Box 430, 40530 Gothenburg, Sweden; 3 Department of Neurosurgery, St. Olav’s Hospital, Olav Kyrres Gate, 7006 Trondheim, Norway; 4 Department of Neurosurgery, Uppsala University Hospital, Uppsala, Sweden; 5 Department of Neuroscience, Section of Neurosurgery, Uppsala University, Uppsala, Sweden; 6 Department of Neurosurgery, Karolinska University Hospital, Solna, Sweden; 7 Department of Clinical Neuroscience, Section of Neurosurgery, Karolinska Institutet, Stockholm, Sweden; 8 Department of Neurosurgery, Copenhagen University Hospital, Rigshospitalet, Copenhagen, Denmark

**Keywords:** Chronic subdural hematoma, Surgical evacuation, Recurrence, Irrigation fluid, Temperature

## Abstract

**Background:**

Chronic subdural hematoma (cSDH) is one of the most common conditions encountered in neurosurgical practice. Recurrence, observed in 5–30% of patients, is a major clinical problem. The temperature of the irrigation fluid used during evacuation of the hematoma might theoretically influence recurrence rates since irrigation fluid at body temperature (37 ^o^C) may beneficially influence coagulation and cSDH solubility when compared to irrigation fluid at room temperature. Should no difference in recurrence rates be observed when comparing irrigation-fluid temperatures, there is no need for warmed fluids during surgery. Our main aim is to investigate the effect of irrigation-fluid temperature on recurrence rates and clinical outcomes after cSDH evacuation using a multicenter randomized controlled trial design.

**Methods:**

The study will be conducted in three neurosurgical departments with population-based catchment areas using a similar surgical strategy. In total, 600 patients fulfilling the inclusion criteria will randomly be assigned to either intraoperative irrigation with fluid at body temperature or room temperature. The power calculation is based on a retrospective study performed at our department showing a recurrence rate of 5% versus 12% when comparing irrigation fluid at body temperature versus fluid at room temperature (unpublished data). The primary endpoint is recurrence rate of cSDH analyzed at 6 months post treatment. Secondary endpoints are mortality rate, complications and health-related quality of life.

**Discussion:**

Irrigation-fluid temperature might influence recurrence rates in the evacuation of chronic subdural hematomas. We present a study protocol for a multicenter randomized controlled trial investigating our hypothesis that irrigation fluid at body temperature is superior to room temperature in reducing recurrence rates following evacuation of cSDH.

**Trials registration:**

ClinicalTrials.gov, ID: NCT02757235. Registered on 2 May 2016.

**Electronic supplementary material:**

The online version of this article (doi:10.1186/s13063-017-2194-y) contains supplementary material, which is available to authorized users.

## Background

A chronic subdural hematoma (cSDH) consists of an accumulation of blood and degraded blood products located between the dura and the brain. cSDHs often expand over time causing progressive neurological symptoms due to brain compression. Symptoms include altered mental state, hemiparesis, headache and, in severe cases, loss of consciousness and even death. Chronic subdural hematomas mainly affect older people due to brain atrophy, an increased tendency to fall and a high prevalence of antithrombotic medications [1, 2].

The incidence of cSDH in individuals over 70 years is approximately 58 per 100,000 per year [3]. This makes cSDH one of the most common conditions requiring neurosurgical treatment.

Symptomatic cSDH can, in the majority of cases, be treated by surgical evacuation, most often performed as surgical evacuation via one or more cranial burr holes [4, 5]. Results from previous studies, including a randomized controlled trial (RCT), have showed that postoperative drainage reduces the recurrence rate [6, 7].

The surgical procedure can be combined with intraoperative irrigation of the subdural space, and although potential benefits with this technique have not yet been ascertained in a controlled manner, there is evidence in favor of intraoperative irrigation [7, 8]. Still, the recurrence of cSDH is a major clinical problem with an estimated recurrence frequency of 3–21.5% with closed-system drainage [9].

The irrigation-fluid temperature may have an impact on recurrence rates of cSDH, possibly due to negative effects on coagulation when using fluid at room temperature [10–12]. It is also plausible that irrigation fluid at body temperature may increase the solubility of the cSDH, thereby facilitating evacuation. The use of body- versus room-temperature irrigation fluid varies between neurosurgical departments, and possibly even between individual surgeons. When a poll on the neurosurgical networking site www.neurosurgic.com addressed this particular question, the result of 620 respondents showed that 57% use irrigation fluid at body temperature and 40% use irrigation fluid at room temperature and 3% did not use any intraoperative irrigation.

The aim of this multicenter randomized controlled study is to investigate whether irrigation-fluid temperature influences clinical outcome and recurrence rates in cSDH.

### Hypothesis

The intraoperative use of irrigation fluid at body temperature results in lower recurrence rates when compared to irrigation fluid at room temperature.

Consequently, the defined null hypothesis will be that there is no difference between the treatment groups.

### Primary aim

The primary aim is to investigate whether irrigation with body-temperature fluid reduces recurrence rates of cSDH compared to room-temperature fluid.

### Secondary aims

Secondary aims include health-related quality of life, complication profile and frequency as well as mortality rate.

## Methods

### Study design

**Fig. 1:**
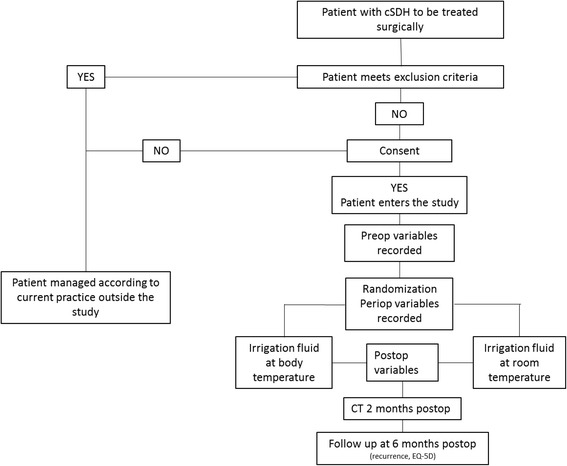
Study flowchart

A multicenter RCT evaluating the use of irrigation fluid at body temperature, 37 ^o^C, versus irrigation fluid at room temperature in burr-hole evacuation of cSDH (Fig. 1). Except from random allocation of treatment between irrigation-fluid temperatures the management of the participants will not differ from the current management of patients treated for cSDH. A 1:1 block randomization will be performed at each clinic.

Participating sites are the Neurosurgical Departments at Sahlgrenska University Hospital (Gothenburg, Sweden), Karolinska University Hospital (Stockholm, Sweden) and the Uppsala University Hospital (Uppsala, Sweden).

### Primary endpoint

The recurrence rate of cSDH within a 6-month follow-up period.

### Secondary endpoints

- Mortality rate
- Complication profile and frequency
- Health-related quality of life at 6 months.
- Neurological function will be compared pre- and postoperatively and registered as improved/in status quo/worsened

The study is registered at ClinicalTrials.gov and reported according to the Standard Protocol Items: Recommendations for Interventional Trials (SPIRIT) guidelines (the SPIRIT Figure and Checklist are available as Additional file 1) for reporting a RCT study protocol [13].

### Study participants

Patients diagnosed with cSDH, where surgical evacuation is indicated, will be screened for inclusion in the study.

#### Inclusion criteria

- Patients with cSDH requiring burr-hole evacuation
- Patients older than 18 years of age

#### Exclusion criteria

- cSDH requiring surgical treatment other than burr-hole evacuation
- cSDH in a patient with an intracranial arachnoidal cyst
- cSDH in a patient with a CSF shunt
- Patients who have undergone intracranial surgery before

Patients with bilateral cSDH will be treated with the same irrigation fluid modality on both sides, and analyzed as a single study participant.

### Informed consent

Informed consent will be obtained from all participants in the study by the attending neurosurgeon prior to surgery of the cSDH. If the patient is unable to give consent, consent will be sought from a close relative. Withdrawal from the study is possible at any time, in accordance with the latest version of the declaration of Helsinki 2013 [14].

### Baseline variables

Variables will be documented in the Case Report Form (CRF) preoperatively, intraoperatively and within 24 h postoperatively by the attending neurosurgeon.

Preoperative variables:

- Age, sex, limb weakness, Glasgow Coma Scale (GCS) score, disorientation, seizures, dysphasia, headache, gait disturbance, mid-line shift, maximal width of the hematoma on computed tomography (CT) and presence of unilateral or bilateral cSDH. Concomitant medication will also be recorded for aspirin, clopidogrel, warfarin and new oral anticoagulants (NOACs). Dexamethasone use will not be recorded since it is not used in the treatment of cSDH in Sweden

Intraoperative variables:

- Unilateral or bilateral surgery, general or local anesthesia, duration of surgery, irrigation fluid volume

Postoperative variables (within 24 h)

- GCS, limb weakness, disorientation, dysphasia, headache, convulsions, gait disturbance, duration of postoperative drainage

### Surgical technique

Current management of cSDH at all participating departments is burr-hole evacuation with intraoperative irrigation followed by active subgaleal drainage as described by Gazerri et al. [15].

The patient will undergo surgery in the supine position under general or local anesthesia, and one to two burr holes are placed over the maximum width of the hematoma. The dura is opened in a cruciate fashion and coagulated by bipolar diathermia. The subdural space is irrigated with Ringer’s lactate at either body- or room temperature according to the result of the randomization. For irrigation, a 50-ml syringe and a soft catheter is used. When the irrigation fluid runs clear a subgaleal drainage is inserted over the burr hole and tunneled away from the skin incision. The drainage is connected to a collection bag with active suction ensuring continuous drainage. The patient is kept in the supine position until the drainage is removed the day after surgery.

If the surgeon decides that a drain cannot be safely inserted the patient will be excluded from the study, although this should be a rare event due to the subgaleal location of the drainage.

### Randomization and blinding

A total of 600 opaque envelopes (200 for each participating centers) with sequential study numbers containing randomly assigned irrigation-fluid temperatures will be prepared. Importantly, the envelopes are not transparent even under direct illumination and will be kept sealed until the patient is draped in the operation room. Also, the envelopes are interconnected so that it is impossible to open these in the wrong order by mistake or on purpose. The envelope will be opened at the time of surgery by the surgeon performing the procedure.

It will not be possible mask the treatment allocation from the treating surgeon; nevertheless, measures will be taken to minimize bias.

1. The patient will not be informed of treatment allocation
2. To minimize detection and selection bias the treatment allocation will not be documented in medical records
3. The investigator performing the statistical analyses will be blinded to treatment assignment until the final analysis is completed

### Outcome measures

1. Recurrence rate of same-sided cSDH requiring surgery within 6 months
2. The EuroQol 5D (version 3 L) (EQ-5D 3 L) is a generic measure of health-related quality of life (HRQL) [16]. The questionnaire contains five questions addressing mobility, self-care, usual activities, pain/discomfort and anxiety/depression. Each question can be answered with “no problem,” “slight problem” or “major problem”
3. Complications within the follow-up period will be assessed using the Landriel Ibanez classification system [17]. This system grades complications in four grades (grade 1: no invasive treatment required; grade 2: invasive treatment required, but not ICU; grade 3: invasive treatment required and intensive care unit (ICU) admission; grade 4: death)
4. Mortality will be analyzed as overall survival 6 months post treatment

### Follow-up

The follow-up period is 6 months. Within this period, recurrences requiring surgery are registered together with any complications and the mortality rate of the participants.

At 2 months after surgery a CT scan is performed to assess any residual hematoma.

At 6 months after surgery HRQL will be assessed using the EQ-5D 3 L questionnaire, mailed to each participant.

Any loss to follow-up will be recorded.

#### Indication for reoperation

This is based on usual clinical practice and is consequently a decision made by the treating physician, often the neurosurgeon who is on-call. The indication for reoperation is typically based on the presence of significant residual or recurrent neurological symptoms (headache, paresis, dysphasia, etc.). If symptoms are present, a CT scan is performed and if there is cSDH causing mass effect correlating with the symptoms the patient will undergo reoperation.

### Sample size calculations

The total number of patients will be 496 (248 in each group, respectively) for a power of 80%. The calculation is based on retrospective pilot study performed at Sahlgrenska University Hospital, 2013–2014, with a 5% versus 12% recurrence rate when comparing irrigation with body-temperature fluid versus room-temperature fluid, respectively (unpublished data). A *p* value of ≤ 0.05 is considered statistically significant.

To compensate for a loss to follow- up, we aim to include a total of 600 patients. Each center will recruit a number of 200 study participants ensuring homogenous recruitment between the participating centers. Although we do not anticipate a large loss to follow-up regarding the primary endpoint we believe that this may be the case for the secondary endpoints. Thus, we will mainly compensate for a loss to follow-up regarding the secondary outcome measures such as EQ-5D 3 L.

### Data management and statistical analysis

A statistician blinded for treatment assignments will perform the analysis according to the intention-to-treat principle.

The primary endpoint (rate of recurrences requiring surgery within 6 months) will be analyzed using the *Χ* ^2^ test for frequency comparison.

Secondary endpoints will be analyzed using appropriate statistics. Categorical data will be compared using *Χ* ^2^ test or Fisher’s exact test. Normally distributed numerical data will be analyzed using *t* test and the Mann-Whitney *U* test if skewed. Log-rank and Kaplan-Meier plots will be used for overall survival.

Data will be collected in the CRF and on password-protected computers. CRF and consent forms are stored in locked rooms at each department. Each study participant’s documents are identified by the randomization number, ensuring confidentiality.

### Data monitoring

An interim analysis will be performed by an independent statistician when each site has 100 subjects with a completed 6-month follow-up. The interim analysis will focus on differences in mortality and recurrence rates (the primary endpoint) between study groups. In addition, each site will cross monitor each other regarding obtained consents and source of clinical data.

### Ethical approval

The study has been approved by the Regional Ethics Committee in Gothenburg, Sweden on 19 January 2016 (reference number: 932-15) (Additional file 2).

## Discussion

To the best of our knowledge this is the first RCT comparing irrigation-fluid temperatures used during surgical evacuation of cSDH. Chronic subdural hematoma is an increasingly common condition affection older people, believed to be linked to demographic changes, extensive use of anticoagulants/anti-platelet therapy and easy access to radiological examination of the brain [18]. The recurrence rate is rather high, with a need for RCTs in an effort to optimize treatment strategies in hope of reducing the recurrence rates. A possible variable influencing the recurrence rate is the temperature of the irrigation fluid used during hematoma evacuation, by affecting solubility of the hematoma and/or coagulation. To test this theory, the current study was initiated.

If a decreased recurrence rate is observed in this multicenter RCT, a new surgical standard of irrigation temperature can be established. If the need of reoperation could be diminished this has obvious gains both for the patient and the health care system.

Strengths of the study include the RCT setup and the involvement of three independent neurosurgical centers in Sweden, covering 60% of the Swedish population; all of these with population-based catchment areas and using almost identical surgical techniques. Also, a digitilized patient chart system ensures easy access, facilitating follow-up. Limitations include the inability to blind the treating surgeon as to the temperature of the irrigation fluid, generating possible bias. Furthermore, although the surgical strategy is very homogeneous among the participating centers, subtle differences in the surgical method between individual surgeons and also between the centers cannot be excluded.

### Trial status

Inclusion of study participants began on 30 March 2016. The study is ongoing.

## Additional files

Additional file 1: SPIRIT Figure. SPIRIT Checklist. (DOC 127 kb)

Additional file 2: Ethics approval from the regional Ethical Review Board of Gothenburg, Sweden. Approved funds of 203,000 SEK from the regional R&D grant. Approved funds of 20,000 SEK from the Hjalmar Svensson research grant. Approved funds of 50,000 SEK from the Edit Jacobsson research grant. (DOC 120 kb)

## Abbreviations

CRF: Case Report Form
cSDH: Chronic subdural hematoma
GCS: Glasgow Coma Scale
HRQL: Health-related quality of life
ICU: Intensive care unit
NOAC: New oral anticoagulant
RCT: Randomized controlled trial
SPIRIT: Standard Protocol Items: Recommendations for Interventional Trials
